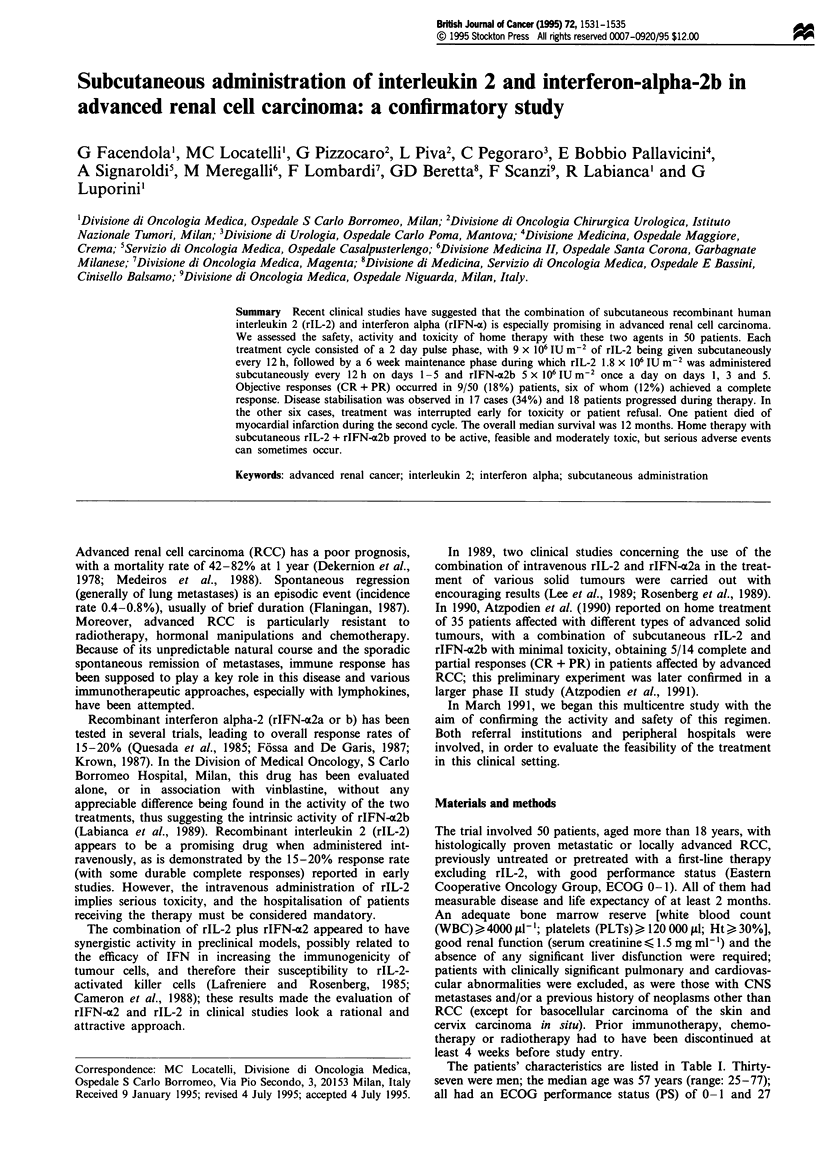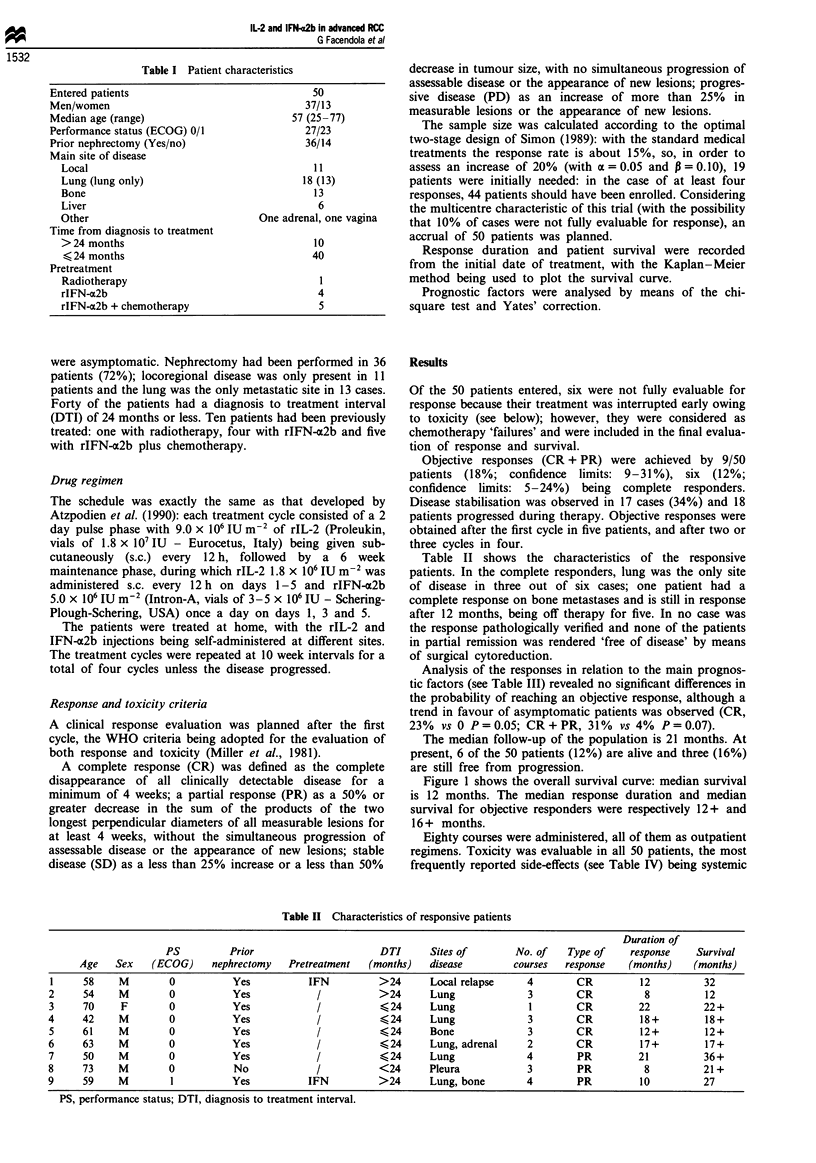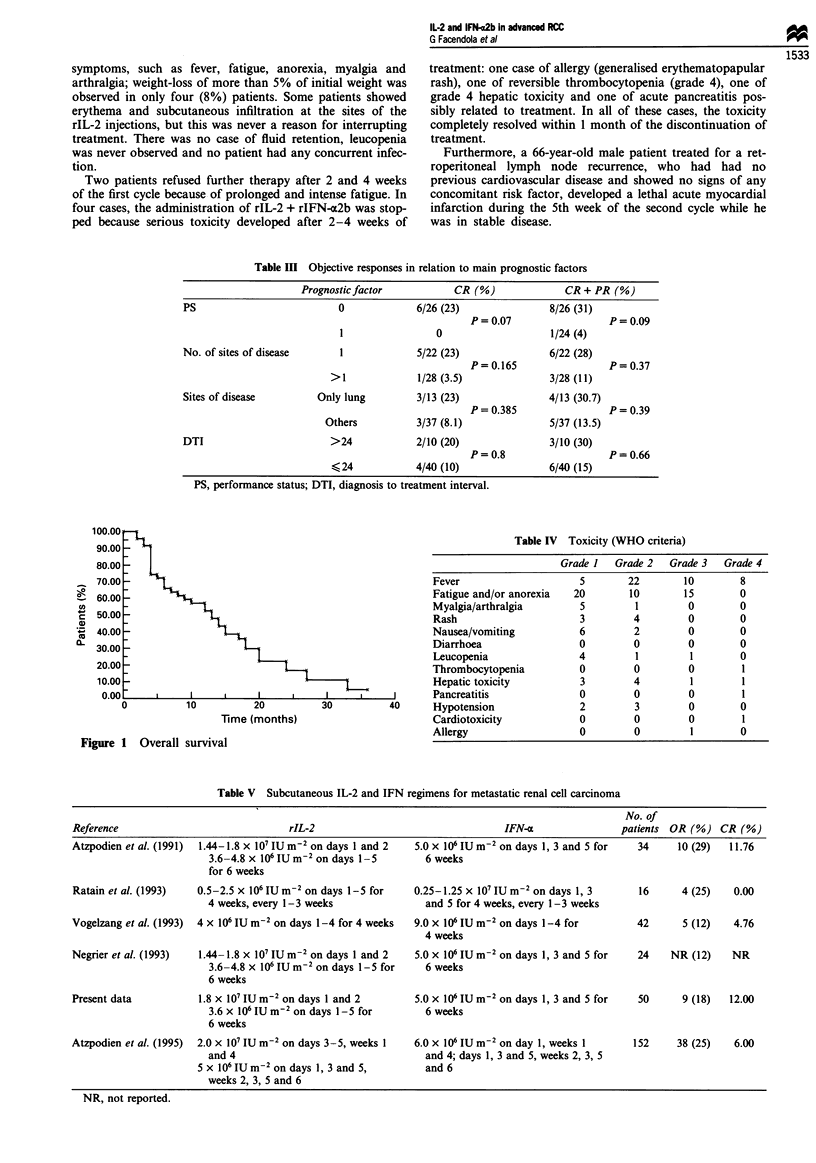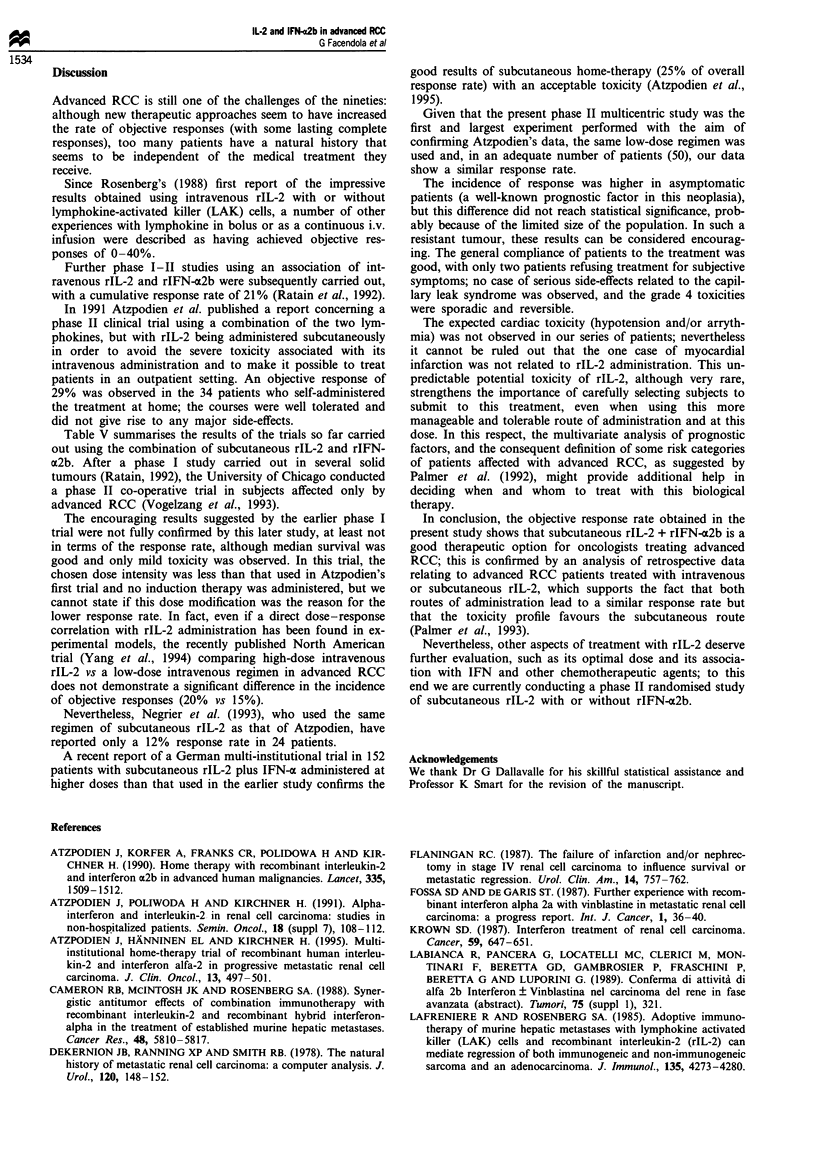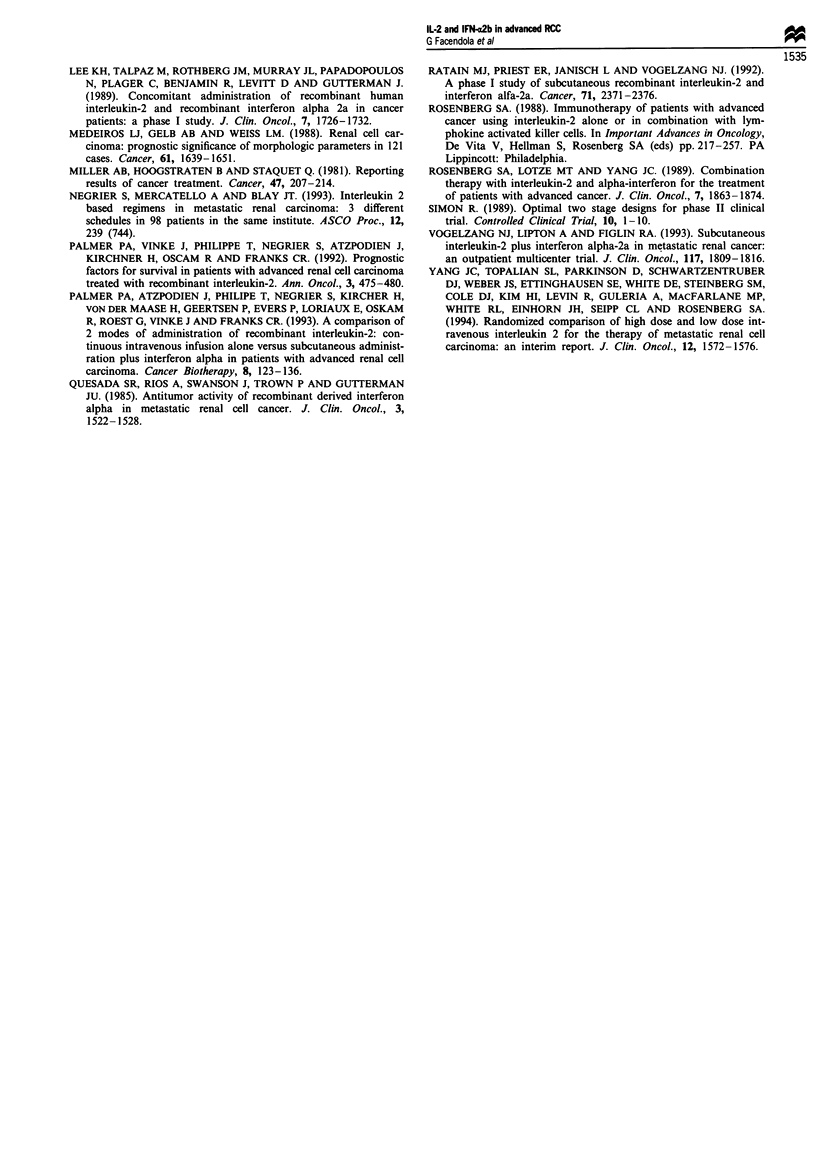# Subcutaneous administration of interleukin 2 and interferon-alpha-2b in advanced renal cell carcinoma: a confirmatory study.

**DOI:** 10.1038/bjc.1995.542

**Published:** 1995-12

**Authors:** G. Facendola, M. C. Locatelli, G. Pizzocaro, L. Piva, C. Pegoraro, E. B. Pallavicini, A. Signaroldi, M. Meregalli, F. Lombardi, G. D. Beretta

**Affiliations:** Divisione di Oncologia Medica, Ospedale S Carlo Borromeo, Milan, Italy.

## Abstract

Recent clinical studies have suggested that the combination of subcutaneous recombinant human interleukin 2 (rIL-2) and interferon alpha (rIFN-alpha) is especially promising in advanced renal cell carcinoma. We assessed the safety, activity and toxicity of home therapy with these two agents in 50 patients. Each treatment cycle consisted of a 2 day pulse phase, with 9 x 10(6) IU m-2 of rIL-2 being given subcutaneously every 12 h, followed by a 6 week maintenance phase during which rIL-2 1.8 x 10(6) IU m-2 was administered subcutaneously every 12 h on days 1-5 and rIFN-alpha 2b 5 x 10(6) IU m-2 once a day on days 1, 3 and 5. Objective responses (CR+PR) occurred in 9/50 (18%) patients, six of whom (12%) achieved a complete response. Disease stabilisation was observed in 17 cases (34%) and 18 patients progressed during therapy. In the other six cases, treatment was interrupted early for toxicity or patient refusal. One patient died of myocardial infarction during the second cycle. The overall median survival was 12 months. Home therapy with subcutaneous rIL-2 + rIFN-alpha 2b proved to be active, feasible and moderately toxic, but serious adverse events can sometimes occur.


British Journal of Cancer (1995) 72, 1531-1535
© 1995 Stockton Press All rights reserved 0007-0920/95 $12.00

### Subcutaneous administration of interleukin 2 and interferon-alpha-2b in advanced renal cell carcinoma: a confirmatory study

G Facendola[1], MC Locatelli[1], G Pizzocaro[2], L Piva[2], C Pegoraro[3], E Bobbio Pallavicini[4], A Signaroldi[5], M Meregalli[6], F Lombardi[7], GD Beretta[8], F Scanzi[9], R Labianca[1] and G Luporini[1]

[1]*Divisione di Oncologia Medica, Ospedale S Carlo Borromeo, Milan;* [2]*Divisione di Oncologia Chirurgica Urologica, Istituto Nazionale Tumori, Milan;* [3]*Divisione di Urologia, Ospedale Carlo Poma, Mantova;* [4]*Divisione Medicina, Ospedale Maggiore, Crema;* [5]*Servizio di Oncologia Medica, Ospedale Casalpusterlengo;* [6]*Divisione Medicina II, Ospedale Santa Corona, Garbagnate Milanese;* [7]*Divisione di Oncologia Medica, Magenta;* [8]*Divisione di Medicina, Servizio di Oncologia Medica, Ospedale E Bassini, Cinisello Balsamo;* [9]*Divisione di Oncologia Medica, Ospedale Niguarda, Milan, Italy.*

**Summary** Recent clinical studies have suggested that the combination of subcutaneous recombinant human interleukin 2 (rIL-2) and interferon alpha (rIFN-α) is especially promising in advanced renal cell carcinoma. We assessed the safety, activity and toxicity of home therapy with these two agents in 50 patients. Each treatment cycle consisted of a 2 day pulse phase, with $9 \times 10^6$ IU $m^{-2}$ of rIL-2 being given subcutaneously every 12 h, followed by a 6 week maintenance phase during which rIL-2 $1.8 \times 10^6$ IU $m^{-2}$ was administered subcutaneously every 12 h on days 1-5 and rIFN-α2b $5 \times 10^6$ IU $m^{-2}$ once a day on days 1, 3 and 5. Objective responses (CR + PR) occurred in 9/50 (18%) patients, six of whom (12%) achieved a complete response. Disease stabilisation was observed in 17 cases (34%) and 18 patients progressed during therapy. In the other six cases, treatment was interrupted early for toxicity or patient refusal. One patient died of myocardial infarction during the second cycle. The overall median survival was 12 months. Home therapy with subcutaneous rIL-2 + rIFN-α2b proved to be active, feasible and moderately toxic, but serious adverse events can sometimes occur.

**Keywords**: advanced renal cancer; interleukin 2; interferon alpha; subcutaneous administration

Advanced renal cell carcinoma (RCC) has a poor prognosis, with a mortality rate of 42-82% at 1 year (Dekernion *et al.*, 1978; Medeiros *et al.*, 1988). Spontaneous regression (generally of lung metastases) is an episodic event (incidence rate 0.4-0.8%), usually of brief duration (Flaningan, 1987). Moreover, advanced RCC is particularly resistant to radiotherapy, hormonal manipulations and chemotherapy. Because of its unpredictable natural course and the sporadic spontaneous remission of metastases, immune response has been supposed to play a key role in this disease and various immunotherapeutic approaches, especially with lymphokines, have been attempted.

Recombinant interferon alpha-2 (rIFN-α2a or b) has been tested in several trials, leading to overall response rates of 15-20% (Quesada *et al.*, 1985; Fössa and De Garis, 1987; Krown, 1987). In the Division of Medical Oncology, S Carlo Borromeo Hospital, Milan, this drug has been evaluated alone, or in association with vinblastine, without any appreciable difference being found in the activity of the two treatments, thus suggesting the intrinsic activity of rIFN-α2b (Labianca *et al.*, 1989). Recombinant interleukin 2 (rIL-2) appears to be a promising drug when administered intravenously, as is demonstrated by the 15-20% response rate (with some durable complete responses) reported in early studies. However, the intravenous administration of rIL-2 implies serious toxicity, and the hospitalisation of patients receiving the therapy must be considered mandatory.

The combination of rIL-2 plus rIFN-α2 appeared to have synergistic activity in preclinical models, possibly related to the efficacy of IFN in increasing the immunogenicity of tumour cells, and therefore their susceptibility to rIL-2-activated killer cells (Lafreniere and Rosenberg, 1985; Cameron *et al.*, 1988); these results made the evaluation of rIFN-α2 and rIL-2 in clinical studies look a rational and attractive approach.

Correspondence: MC Locatelli, Divisione di Oncologia Medica, Ospedale S Carlo Borromeo, Via Pio Secondo, 3, 20153 Milan, Italy
Received 9 January 1995; revised 4 July 1995; accepted 4 July 1995.

In 1989, two clinical studies concerning the use of the combination of intravenous rIL-2 and rIFN-α2a in the treatment of various solid tumours were carried out with encouraging results (Lee *et al.*, 1989; Rosenberg *et al.*, 1989). In 1990, Atzpodien *et al.* (1990) reported on home treatment of 35 patients affected with different types of advanced solid tumours, with a combination of subcutaneous rIL-2 and rIFN-α2b with minimal toxicity, obtaining 5/14 complete and partial responses (CR + PR) in patients affected by advanced RCC; this preliminary experiment was later confirmed in a larger phase II study (Atzpodien *et al.*, 1991).

In March 1991, we began this multicentre study with the aim of confirming the activity and safety of this regimen. Both referral institutions and peripheral hospitals were involved, in order to evaluate the feasibility of the treatment in this clinical setting.

### Materials and methods

The trial involved 50 patients, aged more than 18 years, with histologically proven metastatic or locally advanced RCC, previously untreated or pretreated with a first-line therapy excluding rIL-2, with good performance status (Eastern Cooperative Oncology Group, ECOG 0-1). All of them had measurable disease and life expectancy of at least 2 months. An adequate bone marrow reserve [white blood count (WBC) $\geqslant 4000\ \mu l^{-1}$; platelets (PLTs) $\geqslant 120\,000\ \mu l$; Ht $\geqslant 30\%$], good renal function (serum creatinine $\leqslant 1.5$ mg $ml^{-1}$) and the absence of any significant liver disfunction were required; patients with clinically significant pulmonary and cardiovascular abnormalities were excluded, as were those with CNS metastases and/or a previous history of neoplasms other than RCC (except for basocellular carcinoma of the skin and cervix carcinoma *in situ*). Prior immunotherapy, chemotherapy or radiotherapy had to have been discontinued at least 4 weeks before study entry.

The patients' characteristics are listed in Table I. Thirty-seven were men; the median age was 57 years (range: 25-77); all had an ECOG performance status (PS) of 0-1 and 27

**Table I** Patient characteristics

| | |
|---|---|
| Entered patients | 50 |
| Men/women | 37/13 |
| Median age (range) | 57 (25–77) |
| Performance status (ECOG) 0/1 | 27/23 |
| Prior nephrectomy (Yes/no) | 36/14 |
| Main site of disease | |
| Local | 11 |
| Lung (lung only) | 18 (13) |
| Bone | 13 |
| Liver | 6 |
| Other | One adrenal, one vagina |
| Time from diagnosis to treatment | |
| >24 months | 10 |
| ⩽24 months | 40 |
| Pretreatment | |
| Radiotherapy | 1 |
| rIFN-α2b | 4 |
| rIFN-α2b + chemotherapy | 5 |

were asymptomatic. Nephrectomy had been performed in 36 patients (72%); locoregional disease was only present in 11 patients and the lung was the only metastatic site in 13 cases. Forty of the patients had a diagnosis to treatment interval (DTI) of 24 months or less. Ten patients had been previously treated: one with radiotherapy, four with rIFN-α2b and five with rIFN-α2b plus chemotherapy.

### *Drug regimen*

The schedule was exactly the same as that developed by Atzpodien *et al.* (1990): each treatment cycle consisted of a 2 day pulse phase with $9.0 \times 10^6$ IU $m^{-2}$ of rIL-2 (Proleukin, vials of $1.8 \times 10^7$ IU – Eurocetus, Italy) being given subcutaneously (s.c.) every 12 h, followed by a 6 week maintenance phase, during which rIL-2 $1.8 \times 10^6$ IU $m^{-2}$ was administered s.c. every 12 h on days 1–5 and rIFN-α2b $5.0 \times 10^6$ IU $m^{-2}$ (Intron-A, vials of $3-5 \times 10^6$ IU – Schering-Plough-Schering, USA) once a day on days 1, 3 and 5.

The patients were treated at home, with the rIL-2 and IFN-α2b injections being self-administered at different sites. The treatment cycles were repeated at 10 week intervals for a total of four cycles unless the disease progressed.

### *Response and toxicity criteria*

A clinical response evaluation was planned after the first cycle, the WHO criteria being adopted for the evaluation of both response and toxicity (Miller *et al.*, 1981).

A complete response (CR) was defined as the complete disappearance of all clinically detectable disease for a minimum of 4 weeks; a partial response (PR) as a 50% or greater decrease in the sum of the products of the two longest perpendicular diameters of all measurable lesions for at least 4 weeks, without the simultaneous progression of assessable disease or the appearance of new lesions; stable disease (SD) as a less than 25% increase or a less than 50% decrease in tumour size, with no simultaneous progression of assessable disease or the appearance of new lesions; progressive disease (PD) as an increase of more than 25% in measurable lesions or the appearance of new lesions.

The sample size was calculated according to the optimal two-stage design of Simon (1989): with the standard medical treatments the response rate is about 15%, so, in order to assess an increase of 20% (with $\alpha = 0.05$ and $\beta = 0.10$), 19 patients were initially needed: in the case of at least four responses, 44 patients should have been enrolled. Considering the multicentre characteristic of this trial (with the possibility that 10% of cases were not fully evaluable for response), an accrual of 50 patients was planned.

Response duration and patient survival were recorded from the initial date of treatment, with the Kaplan–Meier method being used to plot the survival curve.

Prognostic factors were analysed by means of the chi-square test and Yates' correction.

### Results

Of the 50 patients entered, six were not fully evaluable for response because their treatment was interrupted early owing to toxicity (see below); however, they were considered as chemotherapy 'failures' and were included in the final evaluation of response and survival.

Objective responses (CR + PR) were achieved by 9/50 patients (18%; confidence limits: 9–31%), six (12%; confidence limits: 5–24%) being complete responders. Disease stabilisation was observed in 17 cases (34%) and 18 patients progressed during therapy. Objective responses were obtained after the first cycle in five patients, and after two or three cycles in four.

Table II shows the characteristics of the responsive patients. In the complete responders, lung was the only site of disease in three out of six cases; one patient had a complete response on bone metastases and is still in response after 12 months, being off therapy for five. In no case was the response pathologically verified and none of the patients in partial remission was rendered 'free of disease' by means of surgical cytoreduction.

Analysis of the responses in relation to the main prognostic factors (see Table III) revealed no significant differences in the probability of reaching an objective response, although a trend in favour of asymptomatic patients was observed (CR, 23% *vs* 0 $P = 0.05$; CR + PR, 31% *vs* 4% $P = 0.07$).

The median follow-up of the population is 21 months. At present, 6 of the 50 patients (12%) are alive and three (16%) are still free from progression.

Figure 1 shows the overall survival curve: median survival is 12 months. The median response duration and median survival for objective responders were respectively 12+ and 16+ months.

Eighty courses were administered, all of them as outpatient regimens. Toxicity was evaluable in all 50 patients, the most frequently reported side-effects (see Table IV) being systemic

**Table II** Characteristics of responsive patients

| | *Age* | *Sex* | *PS (ECOG)* | *Prior nephrectomy* | *Pretreatment* | *DTI (months)* | *Sites of disease* | *No. of courses* | *Type of response* | *Duration of response (months)* | *Survival (months)* |
|---|---|---|---|---|---|---|---|---|---|---|---|
| 1 | 58 | M | 0 | Yes | IFN | >24 | Local relapse | 4 | CR | 12 | 32 |
| 2 | 54 | M | 0 | Yes | / | >24 | Lung | 3 | CR | 8 | 12 |
| 3 | 70 | F | 0 | Yes | / | ⩽24 | Lung | 1 | CR | 22 | 22+ |
| 4 | 42 | M | 0 | Yes | / | ⩽24 | Lung | 3 | CR | 18+ | 18+ |
| 5 | 61 | M | 0 | Yes | / | ⩽24 | Bone | 3 | CR | 12+ | 12+ |
| 6 | 63 | M | 0 | Yes | / | ⩽24 | Lung, adrenal | 2 | CR | 17+ | 17+ |
| 7 | 50 | M | 0 | Yes | / | ⩽24 | Lung | 4 | PR | 21 | 36+ |
| 8 | 73 | M | 0 | No | / | <24 | Pleura | 3 | PR | 8 | 21+ |
| 9 | 59 | M | 1 | Yes | IFN | >24 | Lung, bone | 4 | PR | 10 | 27 |

PS, performance status; DTI, diagnosis to treatment interval.

symptoms, such as fever, fatigue, anorexia, myalgia and arthralgia; weight-loss of more than 5% of initial weight was observed in only four (8%) patients. Some patients showed erythema and subcutaneous infiltration at the sites of the rIL-2 injections, but this was never a reason for interrupting treatment. There was no case of fluid retention, leucopenia was never observed and no patient had any concurrent infection.

Two patients refused further therapy after 2 and 4 weeks of the first cycle because of prolonged and intense fatigue. In four cases, the administration of rIL-2 + rIFN-α2b was stopped because serious toxicity developed after 2–4 weeks of treatment: one case of allergy (generalised erythematopapular rash), one of reversible thrombocytopenia (grade 4), one of grade 4 hepatic toxicity and one of acute pancreatitis possibly related to treatment. In all of these cases, the toxicity completely resolved within 1 month of the discontinuation of treatment.

Furthermore, a 66-year-old male patient treated for a retroperitoneal lymph node recurrence, who had had no previous cardiovascular disease and showed no signs of any concomitant risk factor, developed a lethal acute myocardial infarction during the 5th week of the second cycle while he was in stable disease.

**Table III** Objective responses in relation to main prognostic factors

| | *Prognostic factor* | *CR (%)* | | *CR + PR (%)* | |
|---|---|---|---|---|---|
| PS | 0 | 6/26 (23) | $P = 0.07$ | 8/26 (31) | $P = 0.09$ |
| | 1 | 0 | | 1/24 (4) | |
| No. of sites of disease | 1 | 5/22 (23) | $P = 0.165$ | 6/22 (28) | $P = 0.37$ |
| | >1 | 1/28 (3.5) | | 3/28 (11) | |
| Sites of disease | Only lung | 3/13 (23) | $P = 0.385$ | 4/13 (30.7) | $P = 0.39$ |
| | Others | 3/37 (8.1) | | 5/37 (13.5) | |
| DTI | >24 | 2/10 (20) | $P = 0.8$ | 3/10 (30) | $P = 0.66$ |
| | ⩽24 | 4/40 (10) | | 6/40 (15) | |

PS, performance status; DTI, diagnosis to treatment interval.

**Figure 1** Overall survival

**Table IV** Toxicity (WHO criteria)

| | *Grade 1* | *Grade 2* | *Grade 3* | *Grade 4* |
|---|---|---|---|---|
| Fever | 5 | 22 | 10 | 8 |
| Fatigue and/or anorexia | 20 | 10 | 15 | 0 |
| Myalgia/arthralgia | 5 | 1 | 0 | 0 |
| Rash | 3 | 4 | 0 | 0 |
| Nausea/vomiting | 6 | 2 | 0 | 0 |
| Diarrhoea | 0 | 0 | 0 | 0 |
| Leucopenia | 4 | 1 | 1 | 0 |
| Thrombocytopenia | 0 | 0 | 0 | 1 |
| Hepatic toxicity | 3 | 4 | 1 | 1 |
| Pancreatitis | 0 | 0 | 0 | 1 |
| Hypotension | 2 | 3 | 0 | 0 |
| Cardiotoxicity | 0 | 0 | 0 | 1 |
| Allergy | 0 | 0 | 1 | 0 |

**Table V** Subcutaneous IL-2 and IFN regimens for metastatic renal cell carcinoma

| *Reference* | *rIL-2* | *IFN-α* | *No. of patients* | *OR (%)* | *CR (%)* |
|---|---|---|---|---|---|
| Atzpodien *et al.* (1991) | 1.44–1.8 × $10^7$ IU $m^{-2}$ on days 1 and 2<br>3.6–4.8 × $10^6$ IU $m^{-2}$ on days 1–5 for 6 weeks | 5.0 × $10^6$ IU $m^{-2}$ on days 1, 3 and 5 for 6 weeks | 34 | 10 (29) | 11.76 |
| Ratain *et al.* (1993) | 0.5–2.5 × $10^6$ IU $m^{-2}$ on days 1–5 for 4 weeks, every 1–3 weeks | 0.25–1.25 × $10^7$ IU $m^{-2}$ on days 1, 3 and 5 for 4 weeks, every 1–3 weeks | 16 | 4 (25) | 0.00 |
| Vogelzang *et al.* (1993) | 4 × $10^6$ IU $m^{-2}$ on days 1–4 for 4 weeks | 9.0 × $10^6$ IU $m^{-2}$ on days 1–4 for 4 weeks | 42 | 5 (12) | 4.76 |
| Negrier *et al.* (1993) | 1.44–1.8 × $10^7$ IU $m^{-2}$ on days 1 and 2<br>3.6–4.8 × $10^6$ IU $m^{-2}$ on days 1–5 for 6 weeks | 5.0 × $10^6$ IU $m^{-2}$ on days 1, 3 and 5 for 6 weeks | 24 | NR (12) | NR |
| Present data | 1.8 × $10^7$ IU $m^{-2}$ on days 1 and 2<br>3.6 × $10^6$ IU $m^{-2}$ on days 1–5 for 6 weeks | 5.0 × $10^6$ IU $m^{-2}$ on days 1, 3 and 5 for 6 weeks | 50 | 9 (18) | 12.00 |
| Atzpodien *et al.* (1995) | 2.0 × $10^7$ IU $m^{-2}$ on days 3–5, weeks 1 and 4<br>5 × $10^6$ IU $m^{-2}$ on days 1, 3 and 5, weeks 2, 3, 5 and 6 | 6.0 × $10^6$ IU $m^{-2}$ on day 1, weeks 1 and 4; days 1, 3 and 5, weeks 2, 3, 5 and 6 | 152 | 38 (25) | 6.00 |

NR, not reported.

### Discussion

Advanced RCC is still one of the challenges of the nineties: although new therapeutic approaches seem to have increased the rate of objective responses (with some lasting complete responses), too many patients have a natural history that seems to be independent of the medical treatment they receive.

Since Rosenberg's (1988) first report of the impressive results obtained using intravenous rIL-2 with or without lymphokine-activated killer (LAK) cells, a number of other experiences with lymphokine in bolus or as a continuous i.v. infusion were described as having achieved objective responses of 0–40%.

Further phase I–II studies using an association of intravenous rIL-2 and rIFN-α2b were subsequently carried out, with a cumulative response rate of 21% (Ratain *et al.*, 1992).

In 1991 Atzpodien *et al.* published a report concerning a phase II clinical trial using a combination of the two lymphokines, but with rIL-2 being administered subcutaneously in order to avoid the severe toxicity associated with its intravenous administration and to make it possible to treat patients in an outpatient setting. An objective response of 29% was observed in the 34 patients who self-administered the treatment at home; the courses were well tolerated and did not give rise to any major side-effects.

Table V summarises the results of the trials so far carried out using the combination of subcutaneous rIL-2 and rIFN-α2b. After a phase I study carried out in several solid tumours (Ratain, 1992), the University of Chicago conducted a phase II co-operative trial in subjects affected only by advanced RCC (Vogelzang *et al.*, 1993).

The encouraging results suggested by the earlier phase I trial were not fully confirmed by this later study, at least not in terms of the response rate, although median survival was good and only mild toxicity was observed. In this trial, the chosen dose intensity was less than that used in Atzpodien's first trial and no induction therapy was administered, but we cannot state if this dose modification was the reason for the lower response rate. In fact, even if a direct dose–response correlation with rIL-2 administration has been found in experimental models, the recently published North American trial (Yang *et al.*, 1994) comparing high-dose intravenous rIL-2 *vs* a low-dose intravenous regimen in advanced RCC does not demonstrate a significant difference in the incidence of objective responses (20% *vs* 15%).

Nevertheless, Negrier *et al.* (1993), who used the same regimen of subcutaneous rIL-2 as that of Atzpodien, have reported only a 12% response rate in 24 patients.

A recent report of a German multi-institutional trial in 152 patients with subcutaneous rIL-2 plus IFN-α administered at higher doses than that used in the earlier study confirms the good results of subcutaneous home-therapy (25% of overall response rate) with an acceptable toxicity (Atzpodien *et al.*, 1995).

Given that the present phase II multicentric study was the first and largest experiment performed with the aim of confirming Atzpodien's data, the same low-dose regimen was used and, in an adequate number of patients (50), our data show a similar response rate.

The incidence of response was higher in asymptomatic patients (a well-known prognostic factor in this neoplasia), but this difference did not reach statistical significance, probably because of the limited size of the population. In such a resistant tumour, these results can be considered encouraging. The general compliance of patients to the treatment was good, with only two patients refusing treatment for subjective symptoms; no case of serious side-effects related to the capillary leak syndrome was observed, and the grade 4 toxicities were sporadic and reversible.

The expected cardiac toxicity (hypotension and/or arrythmia) was not observed in our series of patients; nevertheless it cannot be ruled out that the one case of myocardial infarction was not related to rIL-2 administration. This unpredictable potential toxicity of rIL-2, although very rare, strengthens the importance of carefully selecting subjects to submit to this treatment, even when using this more manageable and tolerable route of administration and at this dose. In this respect, the multivariate analysis of prognostic factors, and the consequent definition of some risk categories of patients affected with advanced RCC, as suggested by Palmer *et al.* (1992), might provide additional help in deciding when and whom to treat with this biological therapy.

In conclusion, the objective response rate obtained in the present study shows that subcutaneous rIL-2 + rIFN-α2b is a good therapeutic option for oncologists treating advanced RCC; this is confirmed by an analysis of retrospective data relating to advanced RCC patients treated with intravenous or subcutaneous rIL-2, which supports the fact that both routes of administration lead to a similar response rate but that the toxicity profile favours the subcutaneous route (Palmer *et al.*, 1993).

Nevertheless, other aspects of treatment with rIL-2 deserve further evaluation, such as its optimal dose and its association with IFN and other chemotherapeutic agents; to this end we are currently conducting a phase II randomised study of subcutaneous rIL-2 with or without rIFN-α2b.

**Acknowledgements**
We thank Dr G Dallavalle for his skillful statistical assistance and Professor K Smart for the revision of the manuscript.

### References

ATZPODIEN J, KORFER A, FRANKS CR, POLIDOWA H AND KIRCHNER H. (1990). Home therapy with recombinant interleukin-2 and interferon α2b in advanced human malignancies. *Lancet*, **335**, 1509–1512.

ATZPODIEN J, POLIWODA H AND KIRCHNER H. (1991). Alpha-interferon and interleukin-2 in renal cell carcinoma: studies in non-hospitalized patients. *Semin. Oncol.*, **18** (suppl 7), 108–112.

ATZPODIEN J, HÄNNINEN EL AND KIRCHNER H. (1995). Multi-institutional home-therapy trial of recombinant human interleukin-2 and interferon alfa-2 in progressive metastatic renal cell carcinoma. *J. Clin. Oncol.*, **13**, 497–501.

CAMERON RB, MCINTOSH JK AND ROSENBERG SA. (1988). Synergistic antitumor effects of combination immunotherapy with recombinant interleukin-2 and recombinant hybrid interferon-alpha in the treatment of established murine hepatic metastases. *Cancer Res.*, **48**, 5810–5817.

DEKERNION JB, RANNING XP AND SMITH RB. (1978). The natural history of metastatic renal cell carcinoma: a computer analysis. *J. Urol.*, **120**, 148–152.

FLANINGAN RC. (1987). The failure of infarction and/or nephrectomy in stage IV renal cell carcinoma to influence survival or metastatic regression. *Urol. Clin. Am.*, **14**, 757–762.

FOSSA SD AND DE GARIS ST. (1987). Further experience with recombinant interferon alpha 2a with vinblastine in metastatic renal cell carcinoma: a progress report. *Int. J. Cancer*, **1**, 36–40.

KROWN SD. (1987). Interferon treatment of renal cell carcinoma. *Cancer*, **59**, 647–651.

LABIANCA R, PANCERA G, LOCATELLI MC, CLERICI M, MONTINARI F, BERETTA GD, GAMBROSIER P, FRASCHINI P, BERETTA G AND LUPORINI G. (1989). Conferma di attività di alfa 2b Interferon ± Vinblastina nel carcinoma del rene in fase avanzata (abstract). *Tumori*, **75** (suppl 1), 321.

LAFRENIERE R AND ROSENBERG SA. (1985). Adoptive immunotherapy of murine hepatic metastases with lymphokine activated killer (LAK) cells and recombinant interleukin-2 (rIL-2) can mediate regression of both immunogeneic and non-immunogeneic sarcoma and an adenocarcinoma. *J. Immunol.*, **135**, 4273–4280.

LEE KH, TALPAZ M, ROTHBERG JM, MURRAY JL, PAPADOPOULOS N, PLAGER C, BENJAMIN R, LEVITT D AND GUTTERMAN J. (1989). Concomitant administration of recombinant human interleukin-2 and recombinant interferon alpha 2a in cancer patients: a phase I study. *J. Clin. Oncol.*, **7**, 1726–1732.

MEDEIROS LJ, GELB AB AND WEISS LM. (1988). Renal cell carcinoma: prognostic significance of morphologic parameters in 121 cases. *Cancer*, **61**, 1639–1651.

MILLER AB, HOOGSTRATEN B AND STAQUET Q. (1981). Reporting results of cancer treatment. *Cancer*, **47**, 207–214.

NEGRIER S, MERCATELLO A AND BLAY JT. (1993). Interleukin 2 based regimens in metastatic renal carcinoma: 3 different schedules in 98 patients in the same institute. *ASCO Proc.*, **12**, 239 (744).

PALMER PA, VINKE J, PHILIPPE T, NEGRIER S, ATZPODIEN J, KIRCHNER H, OSCAM R AND FRANKS CR. (1992). Prognostic factors for survival in patients with advanced renal cell carcinoma treated with recombinant interleukin-2. *Ann. Oncol.*, **3**, 475–480.

PALMER PA, ATZPODIEN J, PHILIPE T, NEGRIER S, KIRCHER H, VON DER MAASE H, GEERTSEN P, EVERS P, LORIAUX E, OSKAM R, ROEST G, VINKE J AND FRANKS CR. (1993). A comparison of 2 modes of administration of recombinant interleukin-2: continuous intravenous infusion alone versus subcutaneous administration plus interferon alpha in patients with advanced renal cell carcinoma. *Cancer Biotherapy*, **8**, 123–136.

QUESADA SR, RIOS A, SWANSON J, TROWN P AND GUTTERMAN JU. (1985). Antitumor activity of recombinant derived interferon alpha in metastatic renal cell cancer. *J. Clin. Oncol.*, **3**, 1522–1528.

RATAIN MJ, PRIEST ER, JANISCH L AND VOGELZANG NJ. (1992). A phase I study of subcutaneous recombinant interleukin-2 and interferon alfa-2a. *Cancer*, **71**, 2371–2376.

ROSENBERG SA. (1988). Immunotherapy of patients with advanced cancer using interleukin-2 alone or in combination with lymphokine activated killer cells. In *Important Advances in Oncology*, De Vita V, Hellman S, Rosenberg SA (eds) pp. 217–257. PA Lippincott: Philadelphia.

ROSENBERG SA, LOTZE MT AND YANG JC. (1989). Combination therapy with interleukin-2 and alpha-interferon for the treatment of patients with advanced cancer. *J. Clin. Oncol.*, **7**, 1863–1874.

SIMON R. (1989). Optimal two stage designs for phase II clinical trial. *Controlled Clinical Trial*, **10**, 1–10.

VOGELZANG NJ, LIPTON A AND FIGLIN RA. (1993). Subcutaneous interleukin-2 plus interferon alpha-2a in metastatic renal cancer: an outpatient multicenter trial. *J. Clin. Oncol.*, **117**, 1809–1816.

YANG JC, TOPALIAN SL, PARKINSON D, SCHWARTZENTRUBER DJ, WEBER JS, ETTINGHAUSEN SE, WHITE DE, STEINBERG SM, COLE DJ, KIM HI, LEVIN R, GULERIA A, MACFARLANE MP, WHITE RL, EINHORN JH, SEIPP CL AND ROSENBERG SA. (1994). Randomized comparison of high dose and low dose intravenous interleukin 2 for the therapy of metastatic renal cell carcinoma: an interim report. *J. Clin. Oncol.*, **12**, 1572–1576.